# Low- and High-Intensity Physical Activity Among People with HIV: Multilevel Modeling Analysis Using Sensor- and Survey-Based Predictors

**DOI:** 10.2196/33938

**Published:** 2022-04-14

**Authors:** Paul Cook, Catherine Jankowski, Kristine M Erlandson, Blaine Reeder, Whitney Starr, Mary Beth Flynn Makic

**Affiliations:** 1 College of Nursing University of Colorado Aurora, CO United States; 2 School of Medicine University of Colorado Aurora, CO United States; 3 Sinclair School of Nursing University of Missouri Columbia, MO United States

**Keywords:** ecological momentary assessment, fatigue, HIV, physical activity, stress, mobile phone

## Abstract

**Background:**

High-intensity physical activity improves the health of people with HIV. Even when people have good intentions to engage in physical activity, they often find it difficult to maintain physical activity behavior in the long term. Two Minds Theory is a neurocognitive model that explains gaps between people’s intentions and behaviors based on the operations of 2 independent mental systems. This model predicts that everyday experiences will affect physical activity and that factors outside people’s awareness, such as sleep and stress, can have particularly strong effects on physical activity behaviors.

**Objective:**

We designed this study to test the effects of daily experiences on physical activity among people with HIV, including measures of people’s conscious experiences using daily electronic surveys and measures of nonconscious influences using sensor devices.

**Methods:**

In this study, 55 people with HIV wore a Fitbit Alta for 30 days to monitor their physical activity, sleep, and heart rate variability (HRV) as a physiological indicator of stress. Participants also used their smartphones to complete daily electronic surveys for the same 30 days about fatigue, self-efficacy, mood, stress, coping, motivation, and barriers to self-care. Time-lagged, within-person, multilevel models were used to identify the best prospective predictors of physical activity, considering the daily survey responses of people with HIV and sensor data as predictors of their physical activity the following day. We also tested baseline surveys as predictors of physical activity for comparison with daily variables.

**Results:**

Different people had different average levels of physical activity; however, physical activity also varied substantially from day to day, and daily measures were more predictive than baseline surveys. This suggests a chance to intervene based on day-to-day variations in physical activity. High-intensity physical activity was more likely when people with HIV reported less subjective fatigue on the prior day (*r*=−0.48) but was unrelated to actual sleep based on objective sensor data. High-intensity physical activity was also predicted by higher HRV (*r*=0.56), indicating less stress, lower HIV-related stigma (*r*=−0.21), fewer barriers to self-care (*r*=−0.34), and less approach coping (*r*=−0.34). Similar variables predicted lower-level physical activity measured based on the number of steps per day of people with HIV.

**Conclusions:**

Some predictors of physical activity, such as HRV, were only apparent based on sensor data, whereas others, such as fatigue, could be measured via self-report. Findings about coping were unexpected; however, other findings were in line with the literature. This study extends our prior knowledge on physical activity by demonstrating a prospective effect of everyday experiences on physical activity behavior, which is in line with the predictions of Two Minds Theory. Clinicians can support the physical activity of people with HIV by helping their patients reduce their daily stress, fatigue, and barriers to self-care.

## Introduction

### Background

Physical activity is important in managing many chronic diseases, including HIV. With current antiretroviral treatment (ART) options, people with HIV have almost but not quite a normal life expectancy [1]. Lingering disease-related morbidity and mortality among people with HIV are likely because of accelerated or accentuated aging [2], which may be tied to chronic inflammation and immune activation caused by the virus and by the immune system’s efforts to attack it [3]. In particular, long-term HIV infection predisposes people to develop age-related comorbidities such as heart disease and diabetes at a younger age than their peers without HIV [2].

### Benefits of Physical Activity for People With HIV

Assuming that a patient’s HIV is controlled with ART, prevention of cardiovascular complications in people with HIV involves a number of health promotion strategies similar to those recommended for people without HIV. Hypertension, diabetes, and cholesterol screening are needed, and medications may be required to manage these conditions [4]. Smoking cessation should be discussed, and patients should be supported to quit smoking if desired [5]. Regular exercise, including cardiovascular, strength, flexibility, and balance training, is also recommended. People with HIV, as a group, have less physical activity than that recommended by public health guidelines [6]. In fact, an emerging line of research suggests that people with HIV need more intensive levels of exercise than usual to offset the negative cardiovascular effects of long-term HIV infection [7]. To achieve needed benefits for measures of physical function and cardiovascular fitness, people with HIV require high-intensity physical activity that elevates their heart rate (HR) for at least 30 minutes most days [8]. This is more intensive physical activity than is typical for most Americans [9].

In addition to its cardiovascular benefits, exercise is known to enhance mood and reduce stress, with higher levels of physical activity showing positive effects on many mental health indicators among people with HIV [10]. In addition, exercise interventions have been shown to improve neurocognitive function; improve mitochondrial function; reduce inflammation; improve bone mineral density; reduce fatigue; and, in some studies, directly improve immune function based on CD4 counts among people with HIV [11]. Physical activity may even improve ART medication adherence in people with HIV, an effect that seems to be mediated by reductions in depressive symptoms [12].

### Barriers to Physical Activity Among People With HIV

Unfortunately, depressive symptoms make it less likely for people with HIV to engage in physical activity; other barriers to physical activity among people with HIV include ART side effects, comorbid health problems, physical pain, lower self-efficacy for exercise, fewer perceived benefits of exercise, and lower motivation for exercise [13]. Fatigue is the most common symptom experienced by people with HIV and is cited by patients as a barrier to exercise, although exercise actually tends to reduce fatigue [3]. Qualitative research suggests additional social or environmental barriers to physical activity, including concerns about HIV-related stigma, lack of interpersonal support for exercise, environmental or resource limitations that make physical activity more challenging, and difficulty incorporating exercise into daily activities [14]. Once an exercise habit is started, other barriers to maintenance may arise because the factors that help someone to initiate a physical activity habit are often different from those that help them to maintain it over time [15]; maintenance is more likely when people with HIV have social support and when exercise is well integrated into their daily routines [16].

### Explaining Physical Activity via Two Minds Theory

The differences between the positive effects of exercise and people’s difficulty in adopting and maintaining exercise routines can be explained by Two Minds Theory (TMT), which is a novel approach to understanding and changing health behaviors based on the idea of *intention-behavior gaps* [17]. TMT explains this discrepancy by positing that intentions and behaviors are produced by 2 separate neurocognitive systems. Behavior arises from the *intuitive system*, a set of deep brain structures and functions that perceives situations, compares them to memories, generates emotions, triggers behavioral responses, and is shaped by consequences. All of these intuitive-level processes can occur largely outside of people’s conscious awareness. In contrast, intentions for the future (as well as explanations about past behaviors) are produced by the *narrative system*, a set of conscious higher brain processes coordinated by the prefrontal cortex that interprets and draws conclusions about a person’s experiences and behaviors. The intuitive system is much more efficient and automatic but is more strongly affected by emotions, surface characteristics of a situation, and immediate barriers or rewards; thus, it is sometimes fooled or misled [18]. The narrative–intuitive distinction also helps to explain why, even after a person begins an exercise habit, unexpected barriers can interfere with maintaining it. In general, people start a new behavior because it seems important (ie, when narrative-level benefits are high); however, they continue the new behavior over time when it is easy (ie, when intuitive-level barriers such as HIV-related stigma and environmental challenges are low) [15].

As the intuitive system operates in the moment and in the context of everyday environments, Cook et al [17] suggested that researchers must examine people’s immediate experiences to understand their behavior. Many of the barriers to physical activity for people with HIV fall under the heading of immediate experiences; negative mood, pain, fatigue, ART side effects, and low motivation are all experiential states that go up or down over time for a given person living with HIV [19]. The best practice for studying in-the-moment states is ecological momentary assessment (EMA) using diaries or electronic surveys to collect real-time data on multiple occasions from the same person [20]. Some everyday states are not necessarily accessible for conscious reporting; for example, fatigue may be a symptom experience that people with HIV can report or may manifest via poor sleep, psychomotor slowing, or cognitive confusion [3]. In such cases, ambulatory monitoring data from personal sensor devices may provide additional information [21]. In prior research involving people with HIV, everyday situational factors such as mood, stress, stigma, fatigue, and self-care barriers predicted both ART adherence [19] and fatigue [22]; based on TMT, we expect that these variables would also predict another daily variable, physical activity. Furthermore, we predict that nonconscious influences such as sleep and stress would have particularly strong effects on the subsequent physical activity of people with HIV.

### Purpose of This Study

To better understand the factors that facilitate or interfere with physical activity among people with HIV, we conducted a secondary analysis of data from a mixed method study of daily fatigue experiences among people with HIV [22]. The purpose of the current analysis is to understand the factors that affect everyday physical activity among people with HIV, as measured by 1 month of monitoring using a Fitbit digital sensor device (Fitbit Inc). Predictors of physical activity were assessed using daily sensor data and survey data collected using EMA methods. As EMA generates many days’ worth of data from each individual participant, it permits reliable conclusions to be drawn from studies with smaller sample sizes; the effective *N* for such studies is in between the number of participants and the number of observations after statistically correcting for the dependency of observations within participants.

## Methods

### Recruitment

Recruitment took place in an infectious disease specialty clinic at an academic medical center in Denver, Colorado, United States, from September 2017 to November 2018. The clinic provides care for approximately 1850 people with HIV annually, 97% of whom are on ART, 91% of whom are virally suppressed, and 30% to 45% of whom have significant fatigue [7,23]. The clinic is funded through the Ryan White program and uses a medical home model with on-site services, including mental health, pharmacy, and medical case management. HIV care providers at the clinic screened the patients for potential study eligibility based on the criteria described in the following sections. When a patient was potentially interested, a research team member met with them in the clinic to obtain informed consent. In most cases, the baseline study procedures were completed immediately. In addition, flyers for the study were placed in the clinic waiting room and examination rooms, and patients could self-refer and schedule a time for consent and baseline measures.

The inclusion criteria were people with HIV with (1) well-controlled HIV (viral load<20 copies/mL) with current ART, (2) English language fluency, (3) age of 18 to 80 years, and (4) at least mild fatigue based on the Patient-Reported Outcomes Measurement Information System (PROMIS) 4-item screening tool [24]. The exclusion criteria were (1) serious substance abuse; (2) cognitive impairment; or (3) mental or physical illness that, in the referring clinician’s judgment, precluded participation.

### Procedure

Participants wore a Fitbit Alta HR sensor device for 30 days. The overall physical activity was measured based on the total steps per day, whereas high-intensity physical activity was measured based on the number of *active minutes* when the participant had an elevated HR during exercise. Additional data collected from the Fitbit wristband included sleep metrics, HR indicators, and HR variability (HRV) as a physiological measure of stress.

Participants also completed the validated EMA survey measures on mood, fatigue, self-efficacy, and other psychological variables by responding to a daily message on their smartphones with a link to a REDCap (Research Electronic Data Capture; Vanderbilt University) web-based data form. The EMA survey included questions about situational variables that might interfere with physical activity, such as travel, substance use, or medication side effects.

After consent was obtained, people with HIV completed baseline questionnaires on fatigue and other symptoms. They provided a release of information to extract demographic and clinical data from their electronic health records and received instructions on how to use the Fitbit device and web-based EMA surveys for the next 30 days. A link to complete the survey was delivered once per day at a random time, either by email or SMS text message (participant’s choice). Participants returned after 1 month to complete additional questionnaires and provide a blood sample, and the first 25 participants completed a qualitative interview about their fatigue experiences and physical activity; these data have been presented elsewhere [22].

### Measures

#### Baseline Data

Demographic data from patients’ charts and intake paperwork included age, gender, race, ethnicity, and current employment status. Participants also completed baseline self-report measures, including PROMIS scales for fatigue, depression, applied cognition, sleep, and pain [24]; the HIV Quality of Life scale [25]; and a short form of the HIV Stigma Scale [26].

#### Ambulatory Sensors

The Fitbit wristbands collected continuous ambulatory data on physical activity (total steps and active minutes per day), sleep (time in bed, total sleep time, wake after sleep onset, sleep efficiency, and sleep stages), HR (average, resting, minimum, and maximum), and HRV, with each of the metrics calculated as daily averages for 1 month. Consumer-grade Fitbit monitors provide a balance of sensitivity and data collection efficiency for both activity and sleep data, showing valid step counts compared with research-grade devices and acceptable results for acquiring HRV in field settings [27], as well as high interdevice reliability for various sleep metrics [28]. Fitbit uses a proprietary algorithm to calculate active minutes based on the ratio of current energy expenditure to the amount of energy typically expended by that person at rest [29]; when that ratio is ≥3, Fitbit’s algorithm considers the person to be engaged in high-intensity physical activity [30]. The Fitbit data were retrieved from the manufacturer’s consumer-facing data portal and through an application programming interface [31]. This site allowed the researchers to download participants’ HR data for each minute of the day and then calculate the daily HRV averages for each participant. Participants were allowed to keep the Fitbit device at the conclusion of the study. Participants also used a Pillsy electronic pill bottle to monitor ART adherence (data not presented here) over the same 30 days.

#### Daily Surveys

EMA survey items included the 4-item PROMIS fatigue short form (Cronbach α=.95) [24]; a 4-item self-efficacy scale from the Diary of Ambulatory Behavioral States (DABS) (Cronbach α=.84) [32]; the 3-item DABS mood scale (Cronbach α=.85) [32]; a 6-item stress scale adapted from the Daily Hassles Scale (Cronbach α=.75) [19]; the 9-item Assessment of Daily Coping (Cronbach α=.83), with 2 subscales measuring approach versus avoidance coping [33]; the 2-item DABS social support scale (Cronbach α=.81) [32]; 3 items from the HIV stigma scale (Cronbach α=.77) [26]; the 7-item Herzog motivation scale (Cronbach α=.79) [34]; an ART adherence item from the AIDS Clinical Trials Group measure [35]; and a 9-item barriers to self-care scale developed in our prior daily survey research (Cronbach α=.66), which measured daily situational variables such as travel, substance use, and medication side effects [19]. All reliability statistics reported are from data in this study.

### Statistical Analysis

#### Analysis Plan

To quantify the effects of intuitive-level daily variables versus those of narrative-level measures collected once at baseline, we first examined within-person (day by day) and between-person (stable over time) variation on each of the physical activity measures using graphical displays and intraclass correlation coefficients (*ICCs*). The squared *ICC* is similar to the coefficient of determination (*R^2^*) in regression analysis and can be interpreted as the percentage of variability in the outcome measure that is stable at the level of the individual participant—in other words, the between-person level of variability [36]. Any remaining variability that is *not* accounted for based on the squared *ICC* is the variability that occurs within individuals over time. Some of this remaining variability can potentially be predicted using day-by-day predictor variables that also vary within individuals over time.

We evaluated the effects of stable between-person characteristics using Pearson *r* correlations between participants’ baseline characteristics and each of the 2 physical activity variables. To identify significant correlations, each Pearson *r* was converted to a *t* test using the *r*-to-*t* formula based on sample size, with *P* values obtained from the *t* distribution.

We then tested the effects of within-person sensor and survey variables via within-person multilevel models using SPSS (version 27, IBM Corp). Our models used restricted maximum likelihood estimation to generate β coefficients, which compensated for missing data by imputing a distribution within each participant’s scores based on all available data points. The actual number of survey data points was 58.28% (or 943/1618 person-days) of the possible days. Of the 1618 person-days, data completeness for sensor measures varied by type, with 1130 (69.84%) person-days of valid HR and physical activity data but only 890 (55.01%) person-days of sensor-based sleep data.

A time-lagged analysis was used to predict each day’s physical activity from the experiences of people with HIV on the previous day, which allows for causal conclusions based on the assumption that the cause precedes the effect [23]. Each predictor’s effect on each of the two outcome variables—steps per day as a measure of low- to moderate-intensity exercise and number of active minutes as a measure of high-intensity exercise—was tested separately. For each outcome variable, each possible predictor was tested individually using a minimally restrictive .05 α level. All variables that passed this screening step were then combined into a single multivariable model to identify the most parsimonious set of predictors that together accounted for daily variations in physical activity among people with HIV. In the multivariable models, predictors were entered in block order (HR metrics, then sleep metrics, and then survey data), and backward elimination was used to remove redundant predictors within each block and in the final model.

#### Statistical Power

All models used fixed effects, a standard diagonal matrix, and group mean centering of predictors to control for the clustering of observations within participants. Under these assumptions, a sample size of 55 people with HIV yielded a power of 0.80 to detect moderate effect sizes (β>.40) in multilevel analyses, assuming up to 5 predictors with moderate multicollinearity (variance inflation factor 2.0), moderate *ICC* of 0.50, and α=.05.

### Ethical Considerations

This study was approved by the Colorado Multiple Institutional Review Board (protocol 16-2603).

## Results

### Participant Characteristics

Of the 61 people with HIV approached for the study, 55 (90%) agreed to participate. The most common reasons for nonparticipation were (1) too busy for daily surveys, (2) not interested in using sensor devices, or (3) their smartphones being too old or low on memory to add the software needed for the study. The final sample included 85% (47/55) men and 15% (8/55) women. Participants’ age ranged from 20 to 69 years, and 58% (32/55) of the participants were White and non-Hispanic. In addition, the sample included 20% (11/55) of Black participants and 22% (12/55) of Latino/Latina participants. These demographics are typical of people with HIV in Colorado.

Descriptive analyses of the baseline variables showed high levels of fatigue (greater than the PROMIS 50th percentile on 66% of days; 622/943), a high level of perceived stigma related to HIV (78% of days; 718/921), poor mood (at least once for 80% of people with HIV; 42/52), and high stress (at least once for 53% of people with HIV; 28/52). Furthermore, people with HIV frequently had interrupted sleep based on Fitbit data (284/888, 32% of nights), and some (8/40, 20%) had an overall sleep efficiency level <85%, suggesting disordered sleep. Consistent with the idea that even well-managed HIV involves chronic inflammation, participants had a moderate average C-reactive protein level (mean 2.51, SD 3.34 mg/L), and 5% (2/42) had high levels of inflammation based on C-reactive protein >10. Participant demographics were described in greater detail by Makic et al [22].

### Patterns of Physical Activity

**Figure 1:**
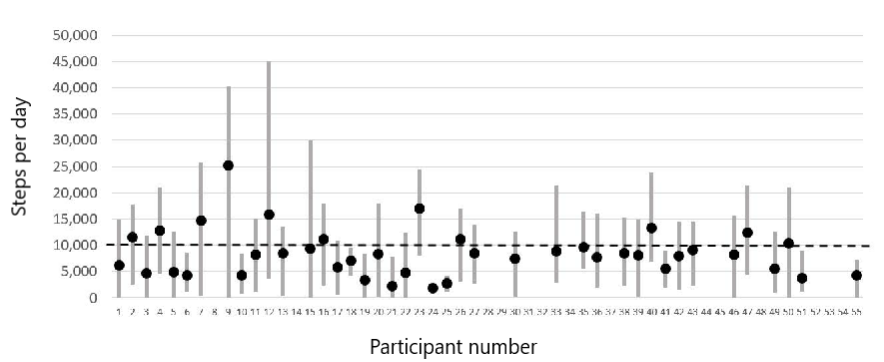
Steps per day for each study participant. Black dots represent each person’s average steps across all days that they wore the Fitbit sensor device (up to 1 month for each participant). Gray bars represent the within-person average (SD 1). SDs were calculated within individuals, and therefore, the varying height of the bars reflects the fact that some people’s daily amount of physical activity varied more than that of others. The dashed line at 10,000 steps reflects the recommended daily amount of overall physical activity for adults.

Physical activity varied dramatically both between and within individuals. Figure 1 shows the average activity levels (black dots) and SDs (gray bars) of the steps-per-day measurements for each participant. As shown in Figure 1, some people with HIV had higher average levels of physical activity than others; however, there was also a high level of within-person variability in physical activity from day to day. The heavy dashed line on the graph illustrates the recommended daily amount of overall physical activity (10,000 steps) for adults; in our sample, 61% (25/44) of participants either met or came close to meeting that goal, which suggests a higher level of physical activity among these people with HIV than among US adults overall. Some individual participants were very active, with an average of ≥15,000 steps per day. The relative length of the bars suggests that participants with lower activity levels were more likely to be consistently inactive; however, among those with high physical activity, there tended to be a broader range of activity from day to day. Overall, the *ICC* for this measure was 0.70, and 51% of the variance (1 – *ICC^2^*) was not explained by a person’s overall tendency toward activity or inactivity. In this context, we looked for within-person predictors that could help explain why some people with HIV exercised more on some days than others.

**Figure 2:**
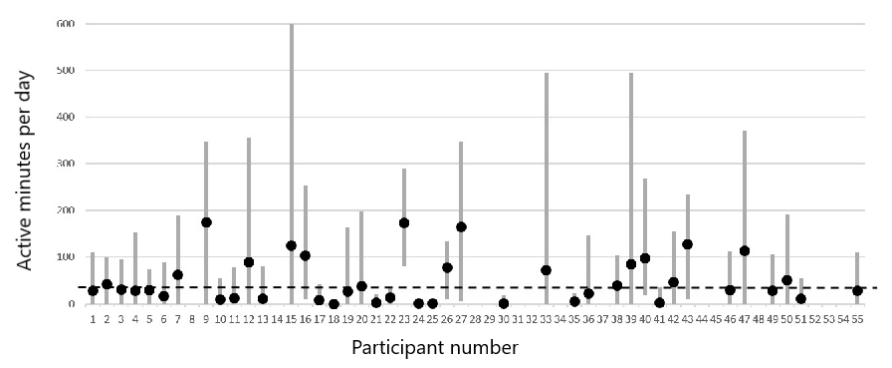
Active minutes per day for each study participant. Black dots represent each person’s average number of active minutes per day, across all days that they wore the Fitbit sensor device (up to 1 month for each participant). Gray bars represent the within-person average (SD 1). SDs were calculated within persons, and therefore, the varying height of the bars reflects the fact that some people’s daily amount of physical activity varied more than that of others. The dashed line at 30 minutes per day reflects the recommended daily amount of active minutes for adults.

Figure 2 shows the second way of conceptualizing physical activity in this study: the number of active minutes per day. This measure may correspond more closely to the high level of physical activity that is needed for people with HIV to prevent cardiovascular complications of HIV infection. Similar to the steps-per-day metric, 59% (26/44) of the participants had cross-day averages above or close to the recommended level of 30 minutes per day. The active minutes measure had a higher *ICC*=0.78, meaning that active minutes were more consistently the same within individuals over time than the total minutes of activity. Only 39% of the variance was unexplained at the between-person level, but given the importance of high activity, and because of the fact that most people with averages <30 minutes per day still had some days with higher activity, it was also important to examine within-person predictors of active minutes.

### Intuitive-Level Predictors of Steps per Day

Table 1 shows the sensor and survey variables that predicted the number of steps taken by the same individual on the subsequent day. People with HIV took more steps the next day when they reported less fatigue, had higher sleep efficiency based on Fitbit sensor data, had higher percentages of time spent in deep sleep and rapid eye movement sleep, and woke up less often after going to bed. Having more steps was also related to lower stress based on higher HRV, although it was not statistically related to self-reported stress. Higher average HR, higher maximum HR, and lower minimum HR values also predicted the number of steps on the subsequent day, although these findings might simply reflect an association between more physical activity and better cardiovascular fitness. Among the survey variables, lower barriers to self-care, more social support, and more use of avoidance coping strategies each predicted the number of steps the following day. The finding on coping was in an unexpected direction and merits further investigation; however, all other relationships were consistent with the literature. In a follow-up exploratory analysis of individual items to determine the specific barriers that were the most important, effects were significant for alcohol use (β=−3311, SE_β_ 875; *P*<.001), drug use (β=1153, SE_β_ 256; *P*<.001), medication side effects (β=−6426, SE_β_ 954; *P*<.001), feeling unwell (β=−1573, SE_β_ 665; *P*=.02), feeling healthy (β=−2038, SE_β_ 660; *P*=.002), and feeling irritated about having to take medications (β=−2428, SE_β_ 621; *P*<.001), but not for forgetting, travel, or feeling confused.

In the multivariable model, the maximum HR and all of the sleep variables were no longer significant. As shown in the right-hand column of Table 1, the final model included average and minimum HR; higher HRV, indicating lower stress; lower self-reported fatigue based on PROMIS; higher self-reported social support; more use of avoidance coping; and fewer reported barriers to self-care.

### Predictors of Active Minutes per Day

A smaller set of daily variables predicted high-intensity physical activity (Table 2). None of the sleep variables had significant effects in univariate analyses. Fewer HR metrics were significant, with higher maximum and average HR predicting active minutes the next day, but no relationship between high-intensity physical activity and minimum HR. Lower stress based on higher HRV predicted more active minutes the next day, and lower self-reported stress also predicted active minutes in a univariate model. In contrast to the findings about total steps, social support was nonsignificant as a predictor of active minutes, but HIV-related stigma (a related measure tied to negative social perceptions rather than positive ones) was significant and predicted active minutes in the opposite direction. Coping was significant in both analyses; however, high-intensity physical activity was predicted by the absence of approach coping rather than by the presence of avoidance coping. This is an inverse coping construct and, therefore, is in the same unexpected direction as the relationship between coping and total steps. The PROMIS fatigue scale and overall barriers to self-care scale again predicted active minutes. In the exploratory analyses, we found the same relationships between individual barriers and active minutes as were seen for total steps; the only exception was alcohol use, which predicted total steps but did not predict active minutes. Self-efficacy also predicted high-intensity physical activity, a relationship that was not seen in the analysis of total steps.

Maximum HR, self-efficacy, and stress were excluded from the multivariable model. Therefore, lower fatigue, less use of approach coping, less HIV-related stigma, and fewer perceived barriers were the only significant EMA predictors of next day active minutes, along with the sensor measures of average HR and lower HRV.

**Table 1:** Predictors of next day total steps.

Daily predictor variable		Univariate model				Combined model		
		β (SE)	*F* test (*df*)	*P* value	β (SE)		*F* test (*df*)	*P* value
**HR^a^ sensor data**								
	Resting HR (bpm^b^)	4.74 (16.0)	0.09 (83)	.77	—^c^		—	—
	Maximum HR (bpm)	33.8 (8.67)	15.2 (520)	<.001	—		—	—
	Average HR (bpm)	133 (15.2)	76.3 (733)	<.001	325 (20.3)		256 (149)	.006
	Minimum HR (bpm)	–45.2 (22.9)	3.88 (520)	.05	–215 (34.9)		38.2 (231)	.01
	HRV^d^ (mspb^e^)	14.5 (4.33)	11.2 (52)	.008	22.8 (5.00)		20.8 (79)	.03
**Sleep sensor data**								
	Total sleep time (minutes)	–1.43 (1.32)	1.18 (473)	.28	—		—	—
	Time in bed (minutes)	–1.19 (1.16)	1.05 (467)	.31	—		—	—
	Sleep efficiency (%)	–7663 (3658)	4.39 (376)	.04	—		—	—
	Interrupted sleep (yes or no)	–719 (377)	3.65 (335)	.06	—		—	—
	Wake after sleep onset (number)	96.9 (47.0)	4.25 (431)	.04	—		—	—
	Light sleep (%)	–449 (1719)	0.07 (98)	.80	—		—	—
	Deep sleep (%)	10,546 (2970)	12.6 (274)	.001	—		—	—
	REM^f^ sleep (%)	–6978 (1423)	24.0 (406)	.03	—		—	—
**Daily survey data**								
	PROMIS^g^ fatigue (*T* score)	–95.9 (9.80)	95.8 (338)	<.001	–46.6 (17.3)		7.25 (260)	.008
	Self-efficacy (1-4 scale)	734 (435)	2.84 (41)	.09	—		—	—
	Mood (1-4 scale)	66.2 (310)	0.05 (161)	.83	—		—	—
	Stress (1-4 scale)	–414 (348)	1.41 (300)	.24	—		—	—
	Avoidance coping (1-4 scale)	1114 (287)	15.1 (295)	.001	964 (244)		15.7 (245)	<.001
	Approach coping (1-4 scale)	589 (336)	3.08 (429)	.08	—		—	—
	Social support (1-4 scale)	822 (278)	871 (445)	.003	530 (254)		4.37 (237)	.04
	Stigma (1-4 scale)	–29.1 (288)	0.01 (355)	.92	—		—	—
	ART^h^ adherence (yes or no)	–526 (1385)	0.14 (134)	.71	—		—	—
	Motivation (1-7 scale)	97.3 (623)	0.02 (367)	.88	—		—	—
	Barriers (1-4 scale)	–3590 (597)	36.1 (221)	<.001	–1396 (514)		7.37 (221)	.008

^a^HR: heart rate.

^b^bpm: beats per minute.

^c^Only statistically significant results are reported.

^d^HRV: heart rate variability.

^e^mspb: milliseconds per beat.

^f^REM: rapid eye movement.

^g^PROMIS: Patient-Reported Outcomes Measurement Information System.

^h^ART: antiretroviral treatment.

**Table 2:** Predictors of next day active minutes.

Daily predictor variable		Univariate model			Combined model		
		β (SE)	*F* test (*df*)	*P* value	β (SE)	*F* test (*df*)	*P* value
**HR^a^ sensor data**							
	Resting HR (bpm^b^)	.26 (.26)	1.03 (528)	.31	—^c^	—	—
	Maximum HR (bpm)	.91 (.10)	82.9 (99)	<.001	—	—	—
	Average HR (bpm)	1.77 (.19)	90.9 (768)	<.001	3.21 (.26)	155 (284)	<.001
	Minimum HR (bpm)	.35 (.26)	1.77 (512)	.18	—	—	—
	HRV^d^ (mspb^e^)	.41 (.05)	55.1 (616)	<.001	.66 (.07)	79.6 (259)	<.001
**Sleep sensor data**							
	Total sleep time (minutes)	–.02 (.02)	0.97 (488)	.33	—	—	—
	Time in bed (minutes)	–.01 (.01)	0.67 (507)	.41	—	—	—
	Sleep efficiency (%)	–41.9 (44.4)	0.89 (536)	.35	—	—	—
	Interrupted sleep (yes or no)	–4.96 (4.67)	1.13 (517)	.29	—	—	—
	Wake after sleep onset (number)	–.20 (.56)	0.13 (205)	.72	—	—	—
	Light sleep (%)	–.01 (.02)	0.09 (98)	.76	—	—	—
	Deep sleep (%)	–.03 (.04)	0.54 (274)	.46	—	—	—
	REM^f^ sleep (%)	–.04 (.06)	0.38 (406)	.54	—	—	—
**Daily survey data**							
	PROMIS^g^ fatigue (*T* score)	–1.81 (.27)	45.4 (185)	<.001	–.98 (0.25)	15.4 (287)	<.001
	Self-efficacy (1-4 scale)	12.2 (5.30)	5.31 (72)	.02	—	—	—
	Mood (1-4 scale)	7.43 (3.85)	3.72 (108)	.06	—	—	—
	Stress (1-4 scale)	–9.45 (4.23)	4.99 (51)	.03	—	—	—
	Avoidance coping (1-4 scale)	1.04 (3.69)	0.08 (165)	.78	—	—	—
	Approach coping (1-4 scale)	–10.4 (4.22)	6.05 (198)	.02	–8.50 (3.77)	5.08 (390)	.03
	Social support (1-4 scale)	6.07 (3.46)	3.09 (226)	.08	—	—	—
	Stigma (1-4 scale)	–9.33 (3.47)	7.24 (50)	.01	–9.59 (3.40)	7.98 (355)	.005
	ART^h^ adherence (yes or no)	–2.27 (19.8)	0.01 (180)	.91	—	—	—
	Motivation (1-7 scale)	.79 (8.39)	0.01 (307)	.93	—	—	—
	Barriers (1-4 scale)	–35.5 (8.03)	19.6 (352)	<.001	–22.4 (7.33)	9.34 (386)	.002

^a^HR: heart rate.

^b^bpm: beats per minute.

^c^Only statistically significant results are reported.

^d^HRV: heart rate variability.

^e^mspb: milliseconds per beat.

^f^REM: rapid eye movement.

^g^PROMIS: Patient-Reported Outcomes Measurement Information System.

^h^ART: antiretroviral treatment.

### Narrative-Level and Demographic Predictors of Physical Activity

Table 3 shows the effects of baseline measures on the 2 physical activity metrics, including demographic variables and self-report questionnaires. These self-report tools assessed some of the same constructs that were included in the daily surveys but did so by engaging the participant’s narrative mind through questions about “average” or “typical” experiences over the past 7 days. Although such retrospective self-report tools are commonly used, prior research suggests that they are more strongly biased by memories, beliefs, and expectancies than in-the-moment questions asked via daily surveys [20].

**Table 3:** Effects of demographic and narrative-level predictors.

Baseline variable		Effect on total steps				Effect on active minutes		
		*r*	*t* test (*df*)	*P* value	*r*		*t* test (*df*)	*P* value
**Demographic predictors**								
	Employed versus not	–0.19	–0.92 (22)	.36	–0.19		-0.93 (22)	.09
	Gender (male or female)	0.003	0.01 (10)	.99	0.28		0.94 (10)	.37
	Race (any minority status)	0.05	0.28 (26)	.78	0.04		0.19 (26)	.85
	Age (years)	0.03	0.23 (54)	.82	0.18		1.42 (54)	.16
**Baseline questionnaires**								
	Fatigue (PROMIS^a^ tool)	0.02	0.15 (54)	.88	–0.15		-1.14 (54)	.26
	Mood (PROMIS tool)	–0.28	–1.92 (54)	.06	–0.28		-2.22 (54)	.03^b^
	Confusion (PROMIS tool)	0.24	1.92 (54)	.06	0.40		3.23 (54)	.002^c^
	Sleep (PROMIS tool)	0.05	0.39 (54)	.70	0.05		0.31 (54)	.76
	Pain (PROMIS tool)	–0.08	–0.61 (54)	.54	–0.03		-0.23 (54)	.82
	Stress (HIV-QoL^d^ subscale)	–0.25	–1.97 (54)	.06	–0.11		-0.81 (54)	.42
	Stigma (HIV Stigma scale)	–0.12	–0.92 (54)	.36	–0.32		-2.66 (54)	.01^b^

^a^PROMIS: Patient-Reported Outcomes Measurement Information System.

^b^Significant at *P*<.05.

^c^Significant at *P*<.01.

^d^HIV-QoL: HIV-related Quality of Life scale.

None of the demographic variables predicted either of the physical activity variables, and none of the baseline questionnaire measures had any effect on the total steps measure of physical activity. Three survey measures predicted active minutes: mood, cognitive confusion, and stigma. Some variables that were significant as day-by-day measures at the intuitive level of analysis failed to predict physical activity when measured at the narrative level, including stress and fatigue. Mood and cognitive confusion had the opposite pattern, with small to moderate effects on active minutes when measured at the narrative level only. Stigma predicted active minutes when measured at either the narrative or intuitive level, although it had a stronger relationship with active minutes when measured day by day.

## Discussion

### Principal Findings

The daily physical activity of people with HIV was predicted based on their previous day environments, experiences, and behaviors. Overall, this study found substantial within-person variability in physical activity and illustrated the importance of everyday experiences and behaviors in understanding daily levels of activity among people with HIV, as suggested by TMT. To our knowledge, this is the first study to examine EMA daily survey measures as predictors of physical activity in people with HIV. Survey-based predictors of overall physical activity in people with HIV included greater avoidance coping, higher perceived social support, and fewer reported barriers to self-care, including the absence of alcohol use, drug use, ART side effects, feeling unwell, and irritation about medication use. The finding on coping was in an unexpected direction; however, other findings were consistent with barriers to physical activity that have been reported in previous studies of people with HIV [13,14].

This study also examined sensor-based predictors of physical activity, which provided data on everyday experiences that might have affected the behavior of people with HIV outside of their conscious awareness. Significant predictors of next day total steps included maximum and average HR, both of which might reflect cardiovascular fitness, as well as HRV, which is a physiological indicator of stress. In contrast to this physiologically based stress metric, participants’ self-reported stress was a less useful predictor that had no association with next day total steps, although it weakly predicted next day active minutes in a univariate model. On the other hand, next day physical activity was more consistently related to subjective fatigue based on the PROMIS tool than to sensor-based measures of sleep quality.

Findings about high-intensity physical activity were generally consistent with those for overall activity, although there were fewer significant predictors for the high-intensity level of physical activity that is more important in preventing cardiovascular disease. High fatigue, high levels of HIV-related stigma, and high stress based on HRV are risk factors for inactivity that can be identified by monitoring everyday psychological states. All of these are potentially modifiable risk factors, which could be addressed by clinicians either directly or with a referral to mental health support services. In a prior study, poor mood was a unique prospective marker for days when people with HIV were less likely to miss a dose of ART medication [19]. Although the mood scale in this study did not significantly predict exercise adherence, related constructs such as stress and fatigue showed significant prospective relationships with next day physical activity.

### Implications for TMT

In this study, variables from EMA surveys and wrist-worn sensors were found to prospectively predict participants’ physical activity on the day after the variable was measured. The daily experiences measured in this study are considered to represent the intuitive system identified by TMT [17], as their closeness in time to the actual point of behavior reduces the chance of retrospective mental editing of experiences. Furthermore, some of the data gathered via the sensors were immune to self-reporting bias and might not even have been accessible using standard retrospective questionnaire methods. Daily within-person variations in behavior accounted for 51% of the total variability in steps per day and 39% of the variability in active minutes, illustrating the importance of testing day-by-day predictors of physical activity. The time-lagged analysis in this study also allowed us to draw prospective conclusions that are stronger than purely correlational findings. Although our results cannot prove a causal relationship because nothing was manipulated, they do show a cause preceding an effect, a feature often lacking in correlational studies.

For a direct comparison between narrative- and intuitive-level predictors, we also examined the effects of stable participant demographic characteristics and the effects of predictor variables assessed using standard retrospective questionnaires at a single point in time. None of these variables were significantly associated with steps per day, and only three of the questionnaire measures—depressed mood, cognitive confusion, and HIV stigma—significantly predicted active minutes. The best narrative-level measures had small to moderate effects, despite the fact that we used best practice PROMIS tools to measure constructs of interest. In general, our findings suggest that intuitive-level measures are more useful than narrative-level measures for predicting the physical activity of people with HIV, although one of the predictors (mood) was significant only when it was measured at the narrative level.

### Strengths and Limitations

Participants in this study were people with HIV recruited from a Ryan White clinic in Denver, Colorado, United States. Of the 1.2 million people currently living with HIV in the United States, approximately 59% are currently in care [37], and of those in care, >80% receive their medical treatment through Ryan White clinics [38]. In that sense, the participants in this study were representative of people with HIV receiving care in the United States. However, our sample was disproportionately White, reflecting the demographics of Colorado, whereas the US population of people with HIV includes higher percentages of people who are Black and Latinx. Therefore, our findings may not be applicable to people of color with HIV.

This study was also limited by a moderate sample size, which may reduce generalizability, although the collection of data on multiple days from each participant increased the effective sample size for multilevel analyses. We used a minimally restrictive α level of .05 to identify potential predictors of physical activity in this initial study of everyday experiences; however, there is a risk of type 1 error in our findings. Future studies with larger sample sizes might use more restrictive statistical assumptions to further narrow the number of predictors of physical activity. In addition, Fitbits are not generally viewed as research-grade devices, although they have shown an ability to differentiate sedentary individuals from moderately active individuals in prior studies. Future research could further clarify the relative importance of predictor variables on physical activity by using research-grade activity sensors that have less measurement error, thereby providing greater statistical power for analyses.

Finally, there may be some measurement challenges in this study: the interpretation of HR metrics, including HRV, is not always clear and may be affected by overall cardiovascular fitness and participants’ psychological states. Other daily measures were self-report surveys, and although the tools were validated in prior EMA studies, there is some potential for bias based on self-presentation or inaccurate recall. Findings on coping were in an unexpected direction, which might also be an indication of measurement problems specific to this construct; for example, a participant’s use of more coping strategies might suggest ineffective or inefficient coping, whereas a high score on just 1 of the 9 items might suggest successful coping. Alternatively, it might be that some participants specifically used exercise as a form of coping; although exercise is a healthy behavior, it could be considered avoidance coping as it does not directly engage with the source of stress. If this is the explanation, it might even be the case that exercising close in time to the source of stress results in more effective coping later on (eg, at a time point that is 2 steps removed from the initial stressor). Thus, the unexpected finding about coping presents intriguing possibilities that could benefit from future research.

### Implications for Practice and Research

Our findings illustrate the need for improved physical activity among people with HIV based on day-to-day variability, even among people with HIV who had average daily activity levels above those recommended by health promotion guidelines. Within-person variability was particularly apparent with regard to high-intensity physical activity. Given that everyday experiences such as stress, fatigue, and HIV-related stigma were found to interfere with high-intensity physical activity, clinicians should ask people with HIV about their stress and fatigue. Clinicians can also help their patients develop strategies to address barriers such as stigma and provide referrals to mental health resources, as appropriate. Finally, clinical care environments should take steps to reduce HIV-related stigma and make people with HIV feel welcomed. This may help to reduce the overall barriers to health care for people with HIV, as well as support their physical activity.

Further research is needed on interventions to increase physical activity among people with HIV. In-the-moment interventions might target improvement in EMA-measured variables such as HIV-related stigma and fatigue, as well as sensor-based predictors such as stress based on HRV. TMT suggests that different variables might be important in initiating versus maintaining physical activity over time; however, this study did not differentiate new versus established exercise habits, which might be another important question to investigate in future intervention studies. Finally, research on physical activity using EMA is still relatively novel, and questions related to the accuracy of measurements using both sensors and surveys are also important topics for ongoing investigation. Future studies using both EMA and sensor data can enhance our understanding of the physical activity and other everyday behaviors of people with HIV.

### Conclusions

Overall, this study suggests an opportunity to improve how people with HIV manage everyday challenges in order to enhance their physical activity. People with HIV reported significant levels of fatigue, which predicted both total steps and high-intensity physical activity. Subjective fatigue was a better predictor of high-intensity physical activity than actual sleep stages, as estimated using the Fitbit device. In addition, stress predicted physical activity; however, a physiological stress measure, HRV, was a stronger predictor of both total steps and active minutes than self-reported daily stress. Other daily psychological experiences, including self-efficacy, coping, and HIV-related stigma, also predicted physical activity, as did everyday self-care barriers such as alcohol use and ART side effects. Many of these are potentially modifiable variables that could be targeted by clinicians or in future research interventions to improve the physical activity of people with HIV.

## Abbreviations

ART: antiretroviral treatment
DABS: Diary of Ambulatory Behavioral States
EMA: ecological momentary assessment
HR: heart rate
HRV: heart rate variability
ICC: intraclass correlation coefficient
PROMIS: Patient-Reported Outcomes Measurement Information System
REDCap: Research Electronic Data Capture
TMT: Two Minds Theory
